# Enhance the delivery of light energy ultra-deep into turbid medium by controlling multiple scattering photons to travel in open channels

**DOI:** 10.1038/s41377-022-00795-8

**Published:** 2022-04-24

**Authors:** Jing Cao, Qiang Yang, Yusi Miao, Yan Li, Saijun Qiu, Zhikai Zhu, Pinghe Wang, Zhongping Chen

**Affiliations:** 1 Beckman Laser Institute, University of California, Irvine, Irvine, CA 92612 USA; 2 Key Laboratory of Biomedical Engineering of Hainan Province, School of Biomedical Engineering, Hainan University, 570228 Hainan, China; 3 Department of Biomedical Engineering, University of California, Irvine, Irvine, CA 92697 USA; 4 China State Key Laboratory of Electronic Thin Films and Integrated Devices, School of Optoelectronic Science and Engineering, University of Electronic Science and Technology of China, 610054 Chengdu, China

**Keywords:** Imaging and sensing, Optical manipulation and tweezers

## Abstract

Multiple light scattering is considered as the major limitation for deep imaging and focusing in turbid media. In this paper, we present an innovative method to overcome this limitation and enhance the delivery of light energy ultra-deep into turbid media with significant improvement in focusing. Our method is based on a wide-field reflection matrix optical coherence tomography (RM-OCT). The time-reversal decomposition of the RM is calibrated with the Tikhonov regularization parameter in order to get more accurate reversal results deep inside the scattering sample. We propose a concept named model energy matrix, which provides a direct mapping of light energy distribution inside the scattering sample. To the best of our knowledge, it is the first time that a method to measure and quantify the distribution of beam intensity inside a scattering sample is demonstrated. By employing the inversion of RM to find the matched wavefront and shaping with a phase-only spatial light modulator, we succeeded in both focusing a beam deep (~9.6 times of scattering mean free path, SMFP) inside the sample and increasing the delivery of light energy by an order of magnitude at an ultra-deep (~14.4 SMFP) position. This technique provides a powerful tool to understand the propagation of photon in a scattering medium and opens a new way to focus light inside biological tissues.

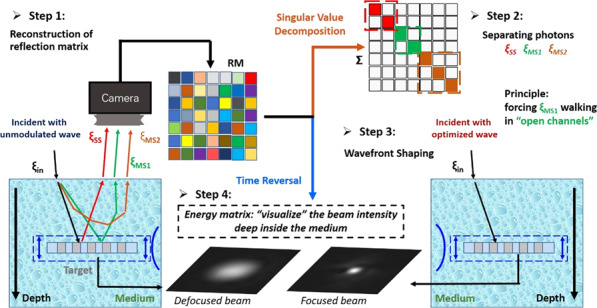

## Introduction

Light scattering is considered a fundamental limitation for deep imaging^1–4^ and focusing^5–8^. There are two reasons, the inhomogeneity of biological tissue and the collision between photons and microscope particles, which lead to the inevitable light scattering events. And the number of scattering events increases with propagation distance. Optical imaging methods can be divided into three groups based on their resolution power. The first is the super-resolution imaging group with resolutions as high as several-tens nanometer^9^. The second is the high-resolution imaging group with resolutions ranging from several microns to dozens of microns. Typical technologies in this group are confocal microscopy^10^, multiple-photons microscopy^11,12^, optical coherence tomography^13^. In general, these technologies rely on the collection of ballistic or single scattering photons. Ballistic photons exist only in the ballistic region, which is very shallow. At the same time, the number of single scattering photons decreases exponentially with penetration depth. As a result, imaging depth achievable by these methods is limited to one transport mean free path (TMFP). Recently, a new concept, depending on the measurement of a sample’s reflection matrix^14^ and time-reversal operator^15^, has demonstrated its ability to separate single scattering photons (arriving the target once and carrying the information of imaging plane) from multiple scattering photons. The particular significance of this technology is the ability to image deeper, about two times more than achievable by optical coherence tomography, without compromising resolution^16,17^. The third group is the low-resolution imaging group with resolution ranging from millimeters to centimeters. These diffused optical imaging technologies rely on the random walk of light. Hence, they usually have deeper imaging capability, but poor spatial resolution.

Scattering events pose limitations not only in imaging but also in beam focusing. Recent advances in optical technology^18^ proved that light scattering could be controlled and beneficial for light focusing^19,20^. By modulating the wavefront of the incident light, one or more focusing points can be achieved inside a scattering medium. For example, the demonstration of wavefront shaping (WFS)^21–23^ and transmission matrix (TM) method^24–27^ show their strengths in focusing light through a highly scattering or even opaque medium^28–30^. These technologies are based on the fact that light scattering is deterministic as long as the sample is motionless. However, the determination of a matched wavefront is a time-consuming process, and the measurement of TM is very complicated. These challenges are significant barriers to overcome prior to the potential translation and application of these technologies. Another option is to use optical phase conjugation^31–34^. This technology can achieve light focusing within a sample in milliseconds. Hence, it is also suitable for dynamic focusing^35–37^. However, it commonly relies on the assistance of ultrasound, two-photon, and fluorescence signals as encoded guide stars^38–40^.

In this paper, we present a novel method to (1) enhance the delivery of light energy ultra-deep into a scattering medium, and to (2) focus light into the sample without any guide-star assistance. Our method is based on a flying spot reflection matrix optical coherence tomography. After obtaining the sample’s RM, the time-reversal operator is calibrated with Tikhonov regularization and deviation criterion to find out those multiple scattering photons that have arrived at the target layer from the entirety of multiple scattering photons. Then with the help of reflection matrix inversion to get the matched wavefronts, a phase-only spatial light modulator is used to generate matched wavefronts to allow these photons to travel in the so-called “open channels”. Open channels enhance the penetration of photons deep into a scattering medium. At the imaging depth of 9.6 times of SMFP, we succeeded in focusing light within the sample. At the ultra-deep position of 14.4 times of SMFP, we succeeded in enhancing intensity of the delivered light by an order of magnitude.

Our method has two advantages. First, we have developed a technique to determine the model energy matrix (E_m_) that helps us “visualizing” the light distribution inside the sample, essentially acting as a camera inserted into the sample to investigate the degree of focus from within. This is the basis of our proposed method, which provides a powerful tool for researching light focusing inside a sample. Second, with the help of E_m_ and wavefront shaping, we have achieved light focusing deep within a scattering medium without guide stars. This has a broad range of applications in the realm of biomedical optics and biomedicine. Furthermore, an optimal wavefront is designed to achieve better focus on all points on the desired target plane inside the scattering media, rather than on only one or a few spots.

## Results

### Principle of controlling multiple scattering photons to travel in open channels

**Fig. 1:**
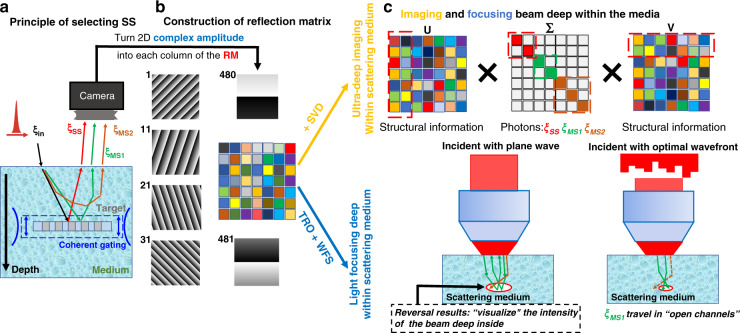
Overview principle of our method to enhance the delivery of light energy deep into the medium. **a** Classification of three types of back-scatted photons, ξ_SS_, ξ_MS1_, and ξ_MS2_ photons. **b** A point-by-point scanning strategy to construct the huge reflection matrix. **c** The theory of (1) time-reversal operator to separate ξ_SS_, ξ_MS1_, and ξ_MS2_ photons; (2) wavefront shaping to focus light within the medium

Figure 1 illustrates a conceptual overview of light focusing deep within a scattering medium. When an ultra-short pulse (ξ_in_) incident beam enters a complex scattering media, there are three types of back-reflected photons. The first is single scattering photons (ξ_SS_), shown using the red arrows in Fig. 1a. These photons carry information of the imaging plane by reaching the target once and then reflected. This type of photons, well known as ballistic photons, is regarded as the ideal means for high-resolution imaging. The second type of photons (ξ_MS1_) are those that also reach the imaging plane but have undergone multiple scattering events (represented by the green arrows in Fig. 1a). These photons carry information from not only the imaging plane but also shallower depths. In general, high-resolution optical imaging methods commonly adopt a time-coherent gating strategy to filter out most of these photons. The third type of photons (ξ_MS2_, illustrated with the brown arrows in Fig. 1a) scatter multiple times and do not reach the imaging plane. However, these photons may have the same time of flight or the light path difference as ξ_SS_, making it impossible to distinguish ξ_MS2_ from ξ_SS_ based on time gating.

We propose a method to further separate the ξ_SS_, ξ_MS1_, and ξ_MS2_ photons from one another. After choosing the ξ_MS1_ photons and forcing them to travel in open channels, we succeeded in focusing a beam deep inside the scattering medium. Our method contains three stages. Stage 1 is the construction of a reflection matrix, which describes the photons (both ξ_SS_ and ξ_MS_) propagation process within the sample. As shown in Fig. 1b, a group of incident wavevectors has covered all the orthogonal input modes for a field of view. Here, we only show the 1st, 11th, 21th, 31th, 480th, and 481th from the entire 961 phase ramps. For each input, the 2D complex amplitude of the interference signal acquired by the camera is reshaped and filled into one column of the RM (see detailed descriptions in “Optical experimental setup of reflection matrix optical coherence tomography”).

After the construction of the RM, which contains abundant information on how light propagates within the sample, a mathematical decomposition, known as time-reversal operator, is applied to the matrix to separate three types of photons from one another. As shown in the upper half of Fig. 1c, $\sum$ is a diagonal matrix with nonzero elements $\delta_{i}$ while $\delta_{1}\,>\, \delta_{2}...\,>\,\delta_{\rm{n}}$. Among all photons, each ξ_SS_ photon experiences only one scattering event, therefore, they are listed at the front of the diagonal. The subsequent part is made out of ξ_MS_ photons, which undergo multiple scattering events.

From the results of our previous work^17^, distinguish ξ_SS_ from ξ_MS_ is enough for ultra-deep imaging. However, with the objective of ultra-deep light focusing in mind, we first need to further separate ξ_MS1_ from the ξ_MS2_ photons. Next, by shaping the wavefront to match the sample, ξ_MS1_ will travel through the sample in the open channels. The photons in these channels will have high transmission coefficient and propagate directly to the desired focusing point, as shown in the lower portion of Fig. 1c. In order to realize this objective, we first introduce the Tikhonov regularization parameter^41^ to calibrate the time-reversal operator (TRO), especially in the deep position (see detailed descriptions in “Model energy matrix to observe the beam intensity of light propagation process within the scattering medium”). On this basis, we propose an energy model matrix to act as a camera inside the sample to “visualize” the intensity of focusing light (see detailed descriptions in “Wavefront shaping based on reflection matrix inversion to focus light within the scattering medium”). Then, RM inversion gives rise to the optimal wavefronts. Finally, by shaping the entire group of incident wavevectors based on the optimal wavefronts, we succeed in enhancing the light energy delivery and beam focusing, ultra-deep inside the scattering medium (see detailed descriptions in “The controlling of ξ_MS2_ photons to travel in “open channels” by wavefront shaping”).

### Optical experimental setup of reflection matrix optical coherence tomography

**Fig. 2:**
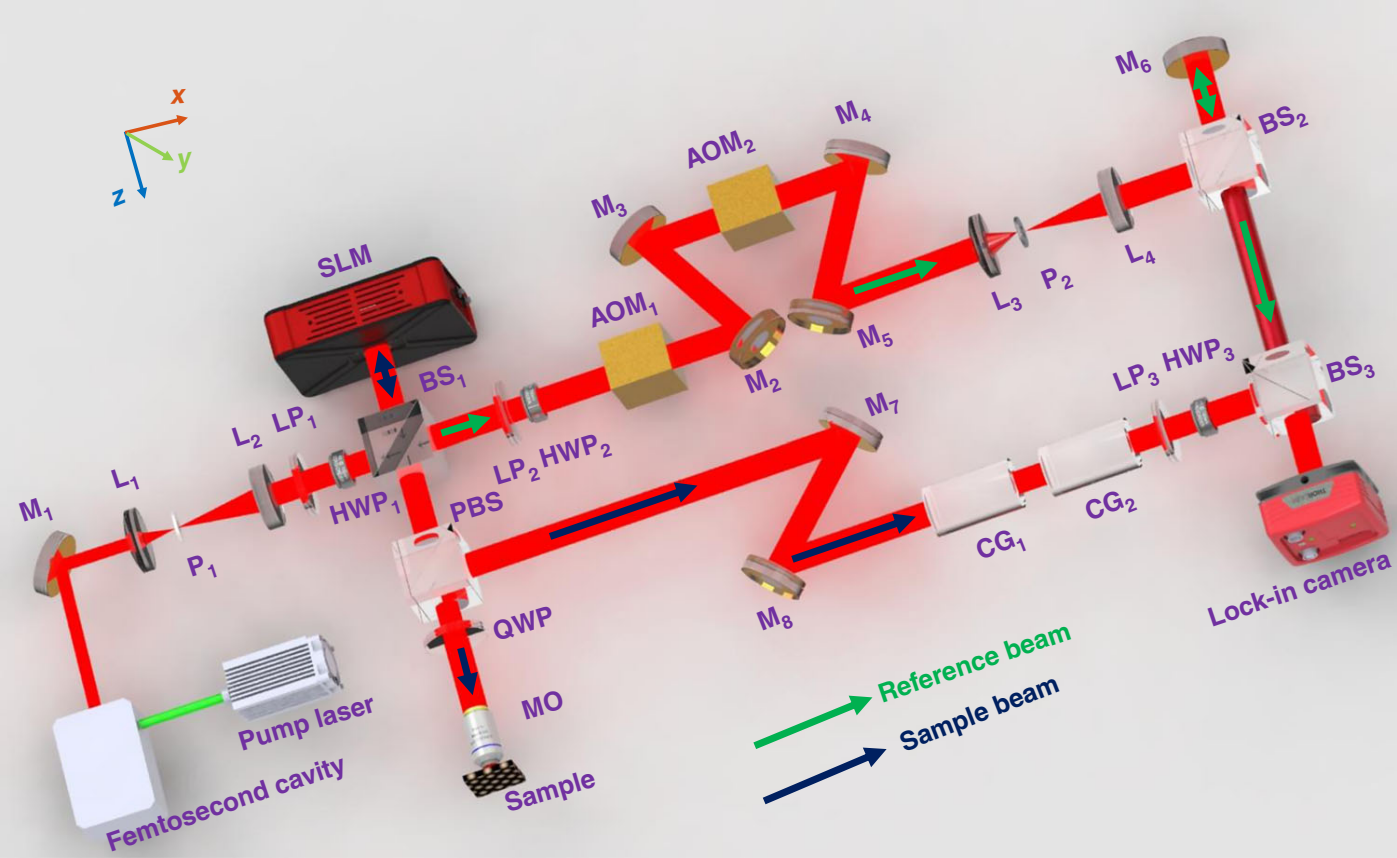
Experimental setup for the wide-field reflection matrix optical coherence tomography to enhance the delivery of light energy deep in the sample. The weak interference signal from mixing the reference arm (green arrow) and sample arm (purple arrow) is first improved using optical heterodyne detection and then recorded by the lock-in camera. M mirror, L lens, P pinhole, LP linear polarizer, HWP half-wave plate, BS beam splitter, PBS polarization beam splitter, AOM acousto-optic modulator, MO microscopy objective, SLM spatial light modulator, CG compensation glass

When light propagates in a turbid medium, the photons inevitably undergo many scattering events. Therefore, any useful signals coming from an ultra-deep position in the sample will be too weak for homodyne detection. Here, we employ heterodyne detection to enhance the detected signal, as shown in Fig. 2. The local oscillator is 80 MHz, which is set by the repetition rate of the ultra-short pulse laser. In our setup, the 40 kHz beat frequency comes from using AOM_1_ operating at +40 MHz and AOM_2_ operating at 40.04 MHz. For signal demodulation, we use a scientific lock-in camera to extract the phase and amplitude of the sample beam. The two advantages of using this advanced CMOS camera are: first, lock-in amplifier technology is especially suitable for our deep imaging setup because of its ability in detecting weak signals; second, in comparison to the conventional four-step phase-shifting method, the camera directly outputs the phase of the sample beam without the need of mechanical movements in the reference arm.

To avoid inaccuracy in the measurement of complex light field caused by the mechanical instability of 2-axis scanning mirrors, we employ a point-to-point scanning strategy. A group of phase maps is loaded onto a phase-only spatial light modulator, to cover a 70 × 70 μm^2^ region of interest. A microscopy objective is then used to collect the back-scattered photons from the illuminated area of the imaging plane. Finally, hundreds of frames of the back-scattered complex light field, each of which corresponds to one focal spot in the focal plane, were combined altogether to generate a 2D reflection matrix. This matrix contains abundant information regarding the position and reflectivity of the targets (detailed descriptions about the experimental setup of RM-OCT are shown in Supplementary materials, Section 1).

### Model energy matrix to observe the beam intensity of light propagation process within the scattering medium

Aiming to analyze the properties of an inverse problem concerning image reconstruction, it is essential to know the light propagation process in a turbid medium. In this paper, we show an experimental method of studying the light-travel process within the sample.

In conventional wavefront shaping experiments, there is always a camera placed behind the sample to monitor the transmitted light. This type of setup suits the purpose of light focusing through the medium. But for the light focusing inside the medium, we propose a model energy matrix (*E*_*m*_) from the above reversal results that acts as a camera inside the sample to “visualize” energy distribution and degree of focus. Equation 1 is the calculation of *E*_*m*_ (a detailed description of how to calibrate the time-reversal operator to get *E*_*m*_ is shown in “Tikhonov regularization method in calibrating the time-reversal operator”).

1 $$E_m = V_JF_JS_JV_J^T$$

**Fig. 3:**
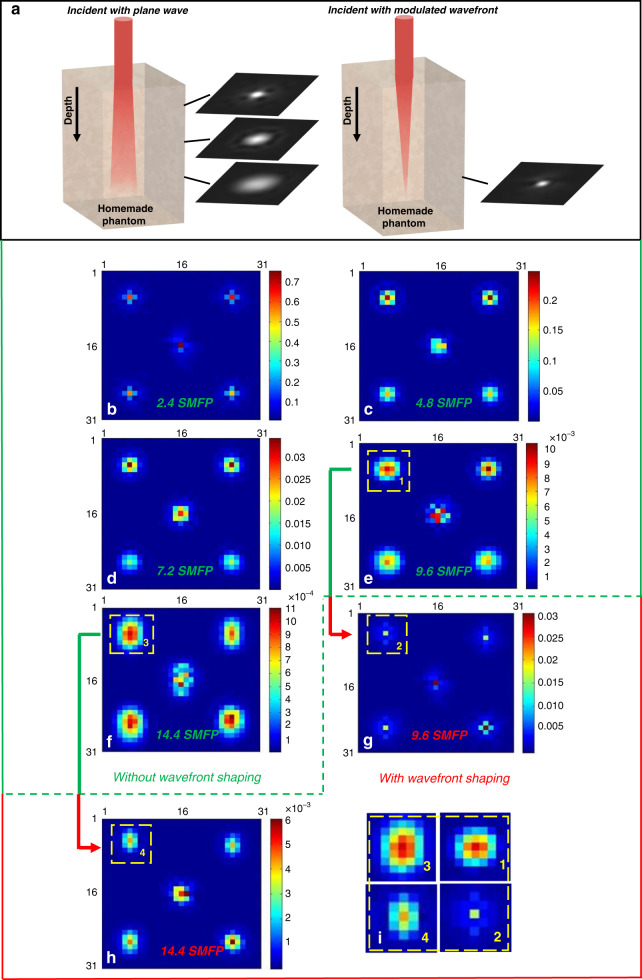
Wavefront shaping helps focus a beam deep inside the homemade phantom. **a** When illuminating the phantom with a plane wave, the beam becomes more defocused with depth; however, if it is illuminated with matched wavefront, the beam can achieve focus even deep inside the phantom. **b**–**f** show the beam intensity patterns at the depths of 2.4, 4.8, 7.2, 9.6, and 14.4 SMFP without wavefront shaping. **g**, **h** show the beam intensity patterns at the depth of 9.6, and 14.4 SMFP when wavefront shaping is employed. **i** shows the magnified views of areas enclosed by yellow dashed lines in (**e**–**h**). The color scale bar is the normalized relative intensity

Figure 3a shows a simplified scheme of beam defocuses as a function of depth when an unmodulated wavefront incident on the phantom; and a beam focusing deep within the same phantom with a matched wavefront. Figure 3b–f shows the distribution of beam energy at five imaging depths, 2.4, 4.8, 7.2, 9.6, and 14.4 SMFP, respectively. To better illustrate the changes in the distribution of light energy and in degree of focus, we look at five scanning points from the entire set of 31 × 31 incident wavevectors, located at (6,6), (6,26), (16,16), (26,6), and (26,26) as examples.

Figure 3b shows the reversal results at superficial depth (2.4 SMFP), where most of the light energy is still precisely concentrated at each of the five incident points. Meanwhile, energy from points adjacent to those five locations amounts to less than 20% of the total energy. This situation is similar to the Airy pattern of a two-dimensional point spread pattern. When the energy distribution meets the following two conditions: (1) the intensity pattern contains a central peak surrounded by a ring with lower intensity; (2) ~80% of the incident intensity is in the central region, we conclude that the beam is focused with diffraction-limitation resolution^42,43^. This usually exists on the surface of the sample when the beam is still focused. Image quality at this depth is aberration-free.

However, when light travels over a long distance within a turbid sample, its energy will redistribute and its degree of focus will degrade due to multiple scattering. Here, we show the experimentally measured reversal results to confirm the above trends. Figure 3c, d shows the reversal results at imaging depths 4.8 and 7.2 SMFP, respectively. For the four incident points located at (6,6), (6,26), (26,6), and (26,26), most of the energy still remains concentrated at each respective position. But for incident point (16,16), the highest energy point has shifted to (16,17) at 4.8 SMFP. Starting from this depth level, the emergence of shifting points means the inaccurate description of the imaging plane (see detailed descriptions in Supplementary materials, Section 2.2). This trend becomes worse as imaging depth increases. In addition, the size of the beam gets larger while the intensity of light decreases, indicating the start of beam defocusing. The above tendencies become even more severe when imaging depth increases to 9.6 and 14.4 SMFP, as shown in Fig. 3e, f.

### Wavefront shaping based on reflection matrix inversion to focus light within the scattering medium

As we know, as a beam passes through a focusing lens, it forms a perfectly focused spot on the surface of a sample. Due to the multiple scattering, transmitted photons with different time-of-flight commonly form a laser speckle pattern which degrades focus. The demonstration of wavefront shaping^44^ proved that multiple scattering can be overcome. Here, we propose a technology with capabilities of light focusing and enhanced energy delivery deep within a scattering medium without any assistance from a guide-star.

Figure 3e shows the reversal beam intensity at 9.6 SMFP when illuminated with a plane wave. As we know, when light travels to the depth of 9.6 SMFP, the ratio of single scattering to multiple scattering photons is ~e^−9.6^. As a result, a beam without its wavefront optimization is highly diverging and its relative intensity is at 10^−3^ level. On the contrary, if the wavefront of a beam is optimized, multiple scattered photons can travel directly to the target, having found their “open channels”. At the same time, the remaining photons will experience a transmission coefficient close to zero; therefore, the relative energy reaching the same depth is enhanced by an order of magnitude while the beam regains its focus (as shown in Fig. 3g).

Figure 3f shows the beam intensity without its wavefront optimization when it traverses a distance of 14.4 SMFP. Compared to its state at the depth of 9.6 SMFP, the beam’s degree of focus degrades more severely at this depth, and attenuation of light intensity further drops to 10^−4^ level. However, after optimizing the wavefront (Fig. 3h), we demonstrated our method’s capability to improve the light energy reaching this depth by an order of magnitude. However, improvement in the light focusing spot size is less significant as compared to that at the depth of 9.6 SMFP. The optimal wavefronts corresponding to the focus at point (6,6), (16,16), and (26,26) are shown in Supplementary materials Section 2.3. Figure 3i shows the magnified views of areas enclosed by yellow dashed lines in Fig. 3e–h.

### The controlling of ξ_MS2_ photons to travel in “open channels” by wavefront shaping

To determine the percentages of ξ_MS1_ photons that have been controlled to travel in open channels, we have calculated the percentages of different types of photons before and after wavefront shaping. The division between ξ_SS_ and ξ_MS_ photons is based on the magnitude of eigenvalues. Photons with eigenvalues lager than the threshold are assumed to be ξ_SS_ photons, while the rest are considered as ξ_MS_ photons.

**Fig. 4:**
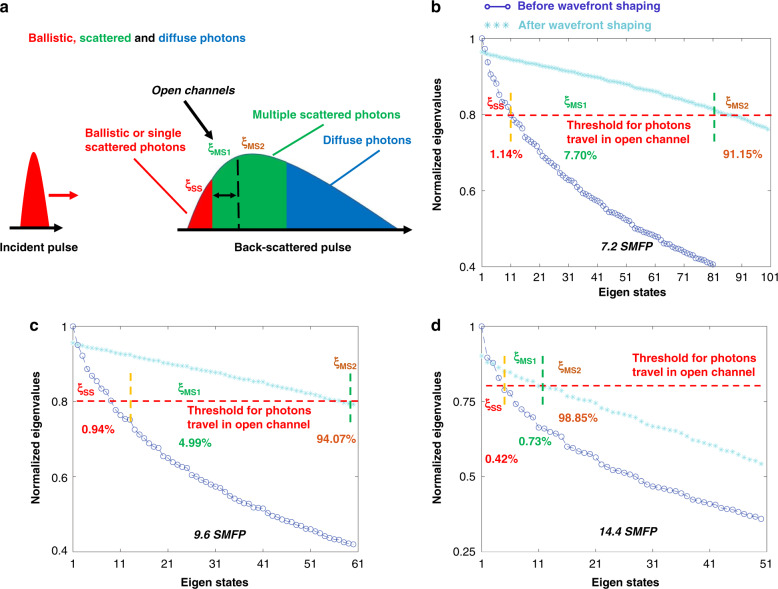
Controlling the ξMS1 photons to travel in open channels. **a** Simple illustration of light pulse reflected from a turbid medium. **b**–**d** show the percentages of ξ_MS1_ photons under control at the depth of 7.2 SMFP, 9.6 SMFP, and 14.4 SMFP, respectively

As illustrated in Fig. 4a, an ultra-short pulse traverses to and from a scattering medium will experience expansion in the time axis. Figure 4b shows the results when the imaging depth is 7.2 SMFP. The purple line with circular markers shows the eigenstates of TRO before wavefront shaping. Based on the principle used to discriminate photons that are traveling in open channels, only 1.14% of photons are ξ_SS_ photons. However, after wavefront shaping, there are 7.70% more photons that have been controlled to travel in open channels. Even so, there are still 91.15% ξ_MS2_ photons, beyond the managing power of our method, which have not been fully modulated. Please note that besides the first 101 eigenstates that we have shown in Fig. 4b, there are a lot of eigenstates (corresponding to ξ_MS2_ photons) that have not been presented.

As one can see in Fig. 4c, d, the amount of ξ_SS_ photons before wavefront shaping decreases to 0.94% (at 9.6 SMFP depth) and 0.42% (at 14.4 SMFP depth), respectively. This explains the phenomenon of beam defocusing along the depth direction, because there is a greater possibility of scattering events and photons changing directions in this process. At the same time, with increasing depth, the percentage of ξ_MS1_ photons, which can be controlled after wavefront shaping, also becomes smaller. There are 4.99% and 0.73% of ξ_MS1_ photons when the imaging depths are 9.6 SMFP and 14.4 SMFP, respectively. Although these numbers are not significant, they provide insights into focusing a beam deep inside a sample, if only we can control more ξ_MS_ photons.

## Discussion

Although scattering is a major challenge in beam focusing inside a turbid medium, we demonstrated our method has the potential to overcome the barriers. By combining reflection matrix measurement, decomposition of time reversal, and wavefront shaping technology, we have presented a systematic method for the investigation of (1) photons propagation process within the sample, (2) filtering single scattering from multiple scattering photons, and (3) controlling photons to focus most of their energy on the target inside a turbid medium.

In addition, we proposed a concept of the model energy matrix, which acts as a camera inside the sample to monitor the beam intensity and degree of focus. We believe our proposed method can further promote the development of wavefront shaping^45–47^. Furthermore, the ability to separate ξ_MS1_ photons that is able to reach the target from the rest of the multiple scattering (ξ_MS2_) photons may also be beneficial for optical imaging methods. Modern advanced optical imaging methods adopt spatial filtering, coherence/time gating, and other strategies to capture the ballistic and single scattering photons to recover the images. But these ξ_MS1_ photons also carry valuable information from the imaging plane. We believe mapping and controlling these photons may pave the way for the emergence of new technologies in light manipulation^48^ and controlling light propagation in a scattering medium^49,50^.

However, some improvements are needed before in vivo applications in biological tissues. One example is the system’s field of view. In our case, we obtain a 70 × 70 μm^2^ region of interest by scanning 31 × 31 points. It currently takes ~1.2 min to measure the reflection matrix. Increasing the number of scanning points helps enlarge the field of view, but at the expense of image acquisition time. Therefore, one main focus is the improvement of the system’s acquisition and post-processing speed. We are now adopting a faster detection technology, replacing the SLM with either a DMD or 1D-SLM for faster modulation speed, and rewriting the Matlab script to employ GPU (graphics-processing unit) processing to accelerate the post-processing algorithm.

In conclusion, we present a method to enhance the delivery of light energy at desired points by an order magnitude at an ultra-deep position of 14.4 SMFP inside the sample. At the same time, we achieved beam focusing at a depth of 9.6 SMFP inside the same medium without any guide-star assistance.

## Materials and methods

### Tikhonov regularization method in calibrating the time-reversal operator

The decomposition of the time-reversal operator to reflection matrix has already proved its effectiveness in distinguishing single scattering photons (ξ_SS_) from multiple scattering photons (ξ_MS_). As for the ultra-deep imaging purpose, the ability to separate out ξ_SS_ is enough. But for the ultra-deep focusing purpose, further separation of ξ_MS1_ from ξ_MS_ is essential before modulating the wavefront to control the transmission mode of ξ_MS1_. Here, we introduce a Tikhonov regularization parameter (*λ*)^41^ to minimize transmission errors during the TRO process. In general, the incident (*E*_in_) and output (*E*_out_) complex light field determine the reflection matrix (*R*) as shown in Eq. 2.

2 $$E_{\mathrm{out}} = R \ast E_{\mathrm{in}}$$

After applying the singular value decomposition (SVD) to *R*, the linear inversion process is shown in Eq. 3. This is also the so-called time-reversal operator, which makes use of the stronger singular values to maximize energy transmission.

3 $$R = U_J \ast S_J \ast V_J^T$$

In Eq. 3, *S*_*J*_ is a diagonal matrix with nonzero singular values *s*_*j*_ in decreasing order. *U*_*J*_ and *V*_*J*_ are orthogonal matrices, which contain the data and spatial information, respectively. *U* = [*u*_*1*_*, u*_*2*_*,…, u*_*m*_] ∈ *R*_*m*_, *V* = [*v*_*1*_*, v*_*2*_*,…, v*_*n*_] ∈ *R*_*n*_, and *S*_*J*_ = diag[*s*_*1*_*, s*_*2*_*,…, s*_*n*_] ∈ *R*_*mn*_. *u*_*j*_ and *v*_*j*_ denote vectors formed by the *j th* columns of *U*_*J*_ and *V*_*J*_.

Compared to *R*, *R*^*†*^*R* is a more standard diagonal matrix with nonzero elements in the diagonal. Thus, the inversion of input-&-output process can be expressed in another way, as shown in Eq. 4.

4 $$R^{\dagger} \ast E_{\mathrm{out}} = R^{\dagger} R \ast E_{\mathrm{in}} = U_JS_J^{ - 1}V_J^T \ast E_{\mathrm{out}}$$

However, this operator is very unstable in the presence of noise. When the aberrant contributions are extremely strong at an ultra-deep position, even the adjacent positions of diagonal in *R*^*†*^*R* emerge a lot of nonzero elements. To reduce the noise influence in describing this inversion process, we introduce *λ* to calibrate the reversal results and to bring them closer to the truths, as shown in Eq. 5.

5 $$E_{\mathrm{in}} = VFS^{\dagger} U^T \ast E_{\mathrm{out}}$$

where *F* is a diagonal matrix containing *λ*. The diagonal elements meet $f_i = s_j^2/\left( {s_j^2 + \lambda ^2} \right)$, which represents the degree of influence to singular matrix *S*_*J*_. In this way, the singular elements with bigger values are kept and calibrated. (See detailed descriptions in Supplementary materials, Section 2.1). Therefore, accurate selection of *λ* becomes a key factor and it is related to imaging depth in our case. Having accurate selection comes at a cost: the more accurate *λ* is, the energy consumption is higher in this process, and the more entropy it produces. Because entropy is inversely correlated with information, higher entropy means there are more possibilities of eigenstates, and the less accurate the description of the time-reversal process becomes. Based on the aiming for less energy loss, we use deviation criterion to scientifically select the most suitable *λ* for different imaging depths^41^. The deviation criterion is estimated by Eq. 6.

6 $$\eta = \min \left( {\frac{{\left\| {e^{est} - e^{mea\_deep}} \right\|}}{{\left\| {e^{deep}} \right\|}}} \right)$$

In Eq. 6, $e^{est} = U_JF_JS_J^{ - 1}V_J^T \ast e^{mea\_surf}$, and it is the evaluated complex electric field with different *λ*. In our case, *λ* ranges from 10^−8^ to 10^8^, interval on logarithmic scale. The *e*^*mea_deep*^ is the measured complex electric field.

### Detailed explanation of experimental procedures

#### Step 1. The measurement of a medium’s RM and model energy matrix with plane waves incident

In our setup, a phase-only spatial light modulator loading with 31 × 31 frames of grating patterns is used to scan the sample for an en face image. The imaging at different depths is obtained by moving a high-precision translation stage attached to the medium. Here, we measured the sample’s RM at five imaging depths (*ξ*_*1*_*-ξ*_*5*_*:* 2,4, 4.8, 7.2, 9.6, and 14.4 SMFP). After the time-reversal operator with calibration has been applied to those RMs, we get the model energy matrix from reversal results to visualize the intensity distributions of beams at five different depths and different positions at each depth. These results are reserved for subsequent comparison to the results after the wavefront is optimized.

#### Step 2. Inversion of RM to get the optimal wavefront

In this step, we need to replace the original plane waves with the 961 matched incident wavefronts. The optimal wavevector $\left( {k\left( {i,\xi } \right),i \in \left( {1:961} \right)} \right)$ for all incident spots is achieved by applying the strategy of inversion of RM. To recover the two-dimensional wavefront of each incident spot, *k (i, ξ)* is reshaped from one dimension to two dimensions (*O (x, y, ξ)*) following the opposite way of how 2D camera data were filled into one column of the RM. This algorithm is based on the theory that RM connects the relationship between the incident light field and output reflected light field. Before loading the optimal phase maps (*O (x, y, ξ)*) onto the SLM, pixel size matching between the camera’s pitch size and SLM’s pitch size is needed. In our case, the camera’s pixel pitch is five times the size of the SLM’s pixel pitch.

#### Step 3. Measurements of the sample’s RM and model energy matrix with renewed optimal wavefronts

This step is a repetition of Step 1. The only difference is that the patterns loading onto the SLM are not initial grating patterns. They have been replaced with the matched wavefronts this time. With wavefront shaping, the ξ_MS1_ would keep the energy when arriving at the designated points. Finally, by comparing the model energy matrix with and without wavefront shaping, it reveals the capability of our method in enhancing the delivery of light energy and focusing light deep inside the sample.

Based on our scanning strategy, the construction of the reflection matrix needs to finish 961 scanning points. The acquisition time for each point is ~80 ms. Therefore, it takes ~76 s each to measure the reflection matrix in Step 1 and Step 3. The post-processing algorithm is written and run in Matlab and it takes ~2 min to get the model energy matrix and matched wavefronts.

### Sample preparation

To demonstrate in a deep scattering region, the effect of the Tikhonov regulation parameter in correcting the decomposition of the time-reversal operator, we designed two sample setups. Sample setup 1: the scattering layer is an A4 paper with a thickness of 97 μm, which corresponds to ~15.2 times of SMFP with the measured SMFP of 5.9 μm. The imaging target is a piece of cover glass with two microbeads on it. Sample setup 2: the scattering layer is a homemade 0.78 mm thickness phantom with 10 g of titanium dioxide in every 100 g of silicone rubber base. The imaging target is a flexible phase-sensitivity material with chemically carved repeating circular patterns.

For the experiments to show the capability of our method in enhancing the delivery of light energy and focusing a beam ultra-deep inside the highly scattering medium, we fabricated a 2 mm-thick phantom with 15 g of titanium dioxide per 100 g of silicone rubber base. By measuring the SMFP of the phantom, the physical thicknesses at 2.4, 4.8, 7.2, 9.6, and 14.4 SMFP are 128, 260, 380, 510, and 760 μm, respectively.

## Supplementary information

Supplementary materials

## Data Availability

The data that support the findings of this study are available from the corresponding author upon reasonable request.
