# Supplementary material for: Enhance the delivery of light energy ultra-deep into turbid medium by controlling multiple scattering photons to travel in open channels
Source: Light Sci Appl. 2022 Apr 24;11:108. doi: 10.1038/s41377-022-00795-8 (PMC9035453; doi:10.1038/s41377-022-00795-8)
Supplement: Supplementary file 1 — Supplementary materials [file 41377_2022_795_MOESM1_ESM.docx]

Supplementary information

**Enhance the delivery of light energy ultra-deep into turbid medium by controlling multiple scattering photons to travel in open channels**

Jing Cao^1,2^, Qiang Yang^1^, Yusi Miao^1,3^, Yan Li^1,3^, Saijun Qiu^1,3^, Zhikai Zhu^1,3^, Pinghe Wang^4,*^, Zhongping Chen^1,3,*^

^1^Beckman Laser Institute, University of California, Irvine, Irvine, California 92612, USA

^2^Key Laboratory of Biomedical Engineering of Hainan Province, School of Biomedical Engineering, Hainan University, Hainan 570228, China

^3^Department of Biomedical Engineering, University of California, Irvine, Irvine, California 92697, USA

^4^China State Key Laboratory of Electronic Thin Films and Integrated Devices, School of Optoelectronic Science and Engineering, University of Electronic Science and Technology of China, Chengdu 610054, China

*Corresponding author: wphsci@uestc.edu.cn, z2chen@uci.edu

**Section. 1 Experimental setup of reflection matrix optical coherence tomography**

The light source is an ultra-short pulse laser (Verdi V-5 Diode-pumped laser, Coherent. Inc & Femtosource compact COCT, Spectra-physics. Inc) with pulse duration ~6 fs, center wavelength ~790 nm, spectrum bandwidth ~90 nm, and output power ~450 mW. For the full use of the SLM’s photosensitive size, the laser beam is expanded by a pair of lenses before going into the low coherence interferometer.

In the reference arm, the frequency of the reference beam shifts with 40 kHz by setting the first acousto-optic modulator (AOM_1_, AOM-405AF3, IntraAction Corp) operating at +40 MHz and the AOM_2_ operating at -40.04 MHz. A pair of lenses and a pinhole are used to collimate and filter out zero-order diffraction beams after the AOMs. The beam successively passes through the beam splitter 2 (BS_2_, 50:50), polarizing beam splitter (PBS_2_, 50:50), and then is reflected by an end mirror, the reference light finally arrives at beam splitter 3 (BS_3_, 50:50). In the sample arm, the beam first illuminated onto a phase-only spatial light modulator (Pluto-2, Holoeye. Inc). A point-by-point scanning strategy is realized by loading a group of desired grating patterns onto the SLM. The reflected beam passed through a maximal reflective light power collection setup, which is made of a PBS_1_ and a QWP with 45 degrees orientation. Successively, a microscope objective (Dry: 25X, 0.25 NA; Water: 40X, 0.80 NA, Olympus. Inc) focuses the light onto the sample. By moving the sample in the z-direction, we can focus the light at different planes. The back-scatted light is collected by the same objective. Two pieces of compensation glass, which is made of the same material as the AOM, have been used to match the dispersion introduced by the two AOMs in the reference arm.

In the detection part, we have combined both optical heterodyne detection and lock-in amplitude technology to precisely obtain useful signals. The interference signal is captured and demodulated by a lock-in camera (Helicam C3, Heliotis. Inc) to directly output two components: phase and amplitude of the sample beam. This camera is based on a proprietary CMOS image sensor where every pixel can acquire and process the optical signals in parallel. Each pixel of the image sensor features an electronic circuit that performs real-time analog preprocessing.

**Section. 2 Detailed descriptions of the TRO calibration process**

**2.1 The effect of Tikhonov regulation parameter (*λ*) in correcting decomposition of the time reversal operator in deep scattering region**

We show the calibration of the time reversal process [1,2] has profound meaning in getting more accurate and closer to true results especially at ultra-deep positions. At the superficial layers, most of the photons only go through a single scattering event. Therefore, the reflection matrix in this situation can describe the sample in a very exact way. This is obvious by observing the distribution of singular values after decomposition of time reversal (DOTR). Non-zero elements only exist in the diagonal, which also means the reversal results truly represent the actual situation. Moreover, it is easy to distinguish those single scattering photons which carry high-order momentum and useful imaging information. Due to the strong aberration, things are quite different in the deep scattering region. One obvious difference is the distribution of singular values. Other than the diagonal elements, many adjacent positions also come up with non-zero elements. A reason for this phenomenon is the inaccuracy measurement of the reflection matrix at ultra-deep penetration depth. Meanwhile, the emerging of these non-zero non-diagonal elements becomes a misguided factor when choosing the first N singular values and their vectors to reconstruct the target. In this circumstance, either for the scientific filtering out single scattering photon(s) purpose or making sure the reversal results closer to the true results, modifying the decomposition of measured with error RM seems to be a necessary process. Here, we show two experimental results of how η corrects the process of DOTR in the deep scattering region.

(1) At multiple scattering regions.


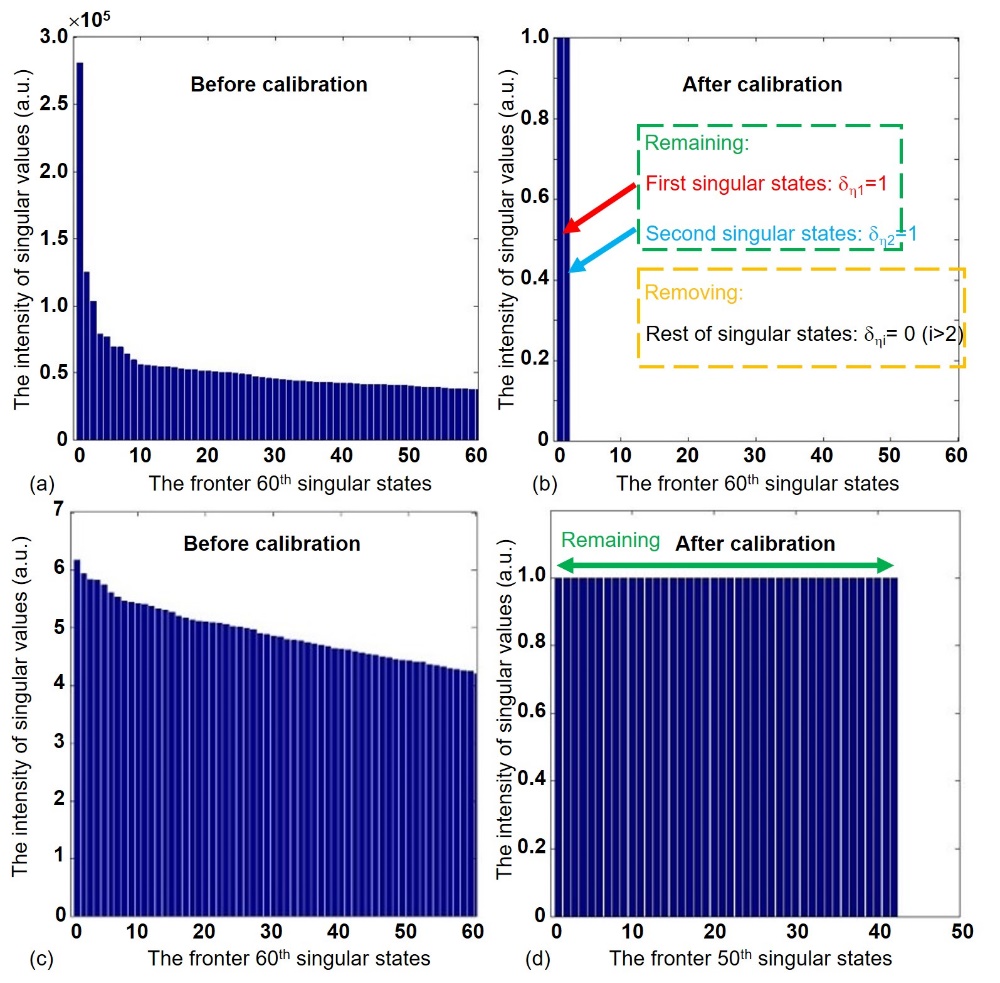


FIG.S1. The effect of Kirchhoff regularization modulation in scientific choosing the eigenstates corresponding to single scattering contributions.

Fig. S1 shows the distribution of singular values before correcting. They are in decreasing order as usual. In principle, the magnitude of singular values represents the energy of photons relatively. The photons that scatter only once usually have less energy loss. As a result, they have strong magnitude and correspond to the front singular values. As a result, these singular values correspond to the multiple scattering photons. From Fig. S1 (a), there is no clear boundary to distinguish single from multiple scattering events. However, after Kirchhoff regularization modulation, a scientific choosing result is demonstrated as shown in Fig. S1 (b). The sample setup is a cover glass attached with two beads beneath a highly scattering layer (A4 paper with ~15.2 SMFP). And we can see the choosing result is in accord with our previous results [3], the first two singular states are related to the two single scattering events that are enough to recover the imaging target.

(2) Deep in biological sample.

The scattering layer is a homemade 0.78 mm phantom. Based on our previous experience, considering the front 38th singular states are enough to reconstruct the imaging target [4]. Nevertheless, this number is known by experience. From the Fig. S1 (c) and (d), with the help of regulation parameter, it recognizes the front 42th states are singles scattering events. And this is so closer to our previously empirical number. Fig.S2 are the diagonal matrix(s) before (a) and after (b) scientific choosing.


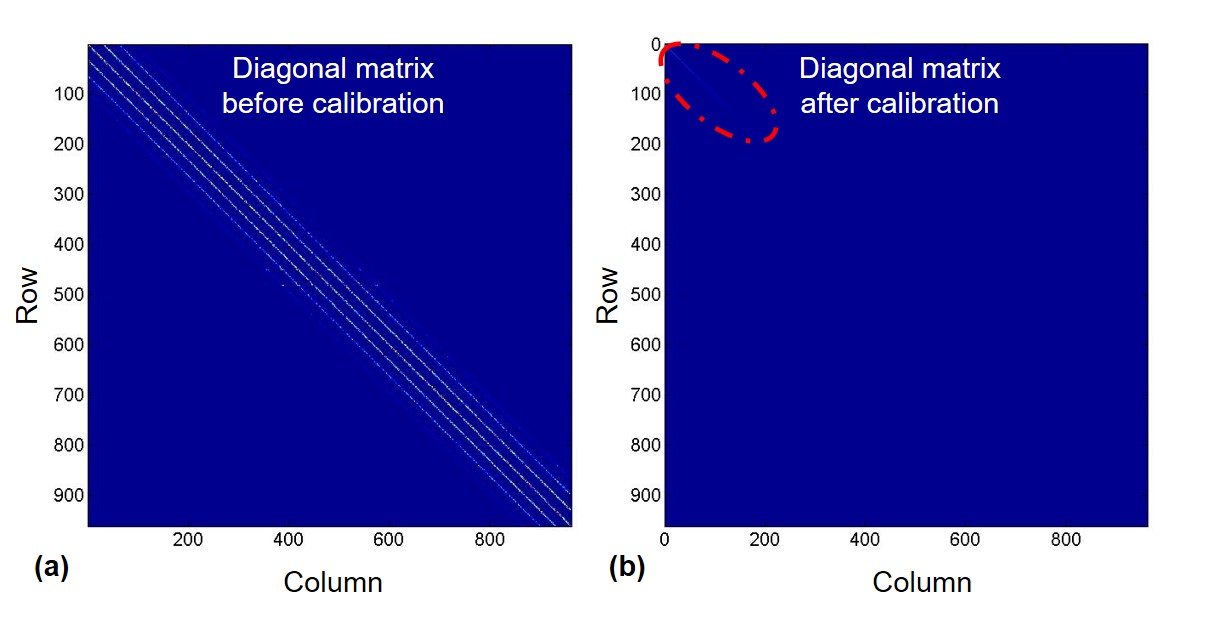


FIG. S2. Comparison of the diagonal matrix before (a) and after (b) calibration.

**2.2 The accuracy rate analysis of scanning beams located at their local positions before and after calibration.**

FIG.S3 shows effect of *λ* in calibrating the maximal intensity of the beams to their located positions. Before calibration, this number is 956, 824, 728, and 516 at the depth of 2.4 SMFP, 4.8 SMFP, 7.2 SMFP, and 9.6 SMFP, respectively. But after calibration, this number increases to 961,914, 875, and 736, respectively.


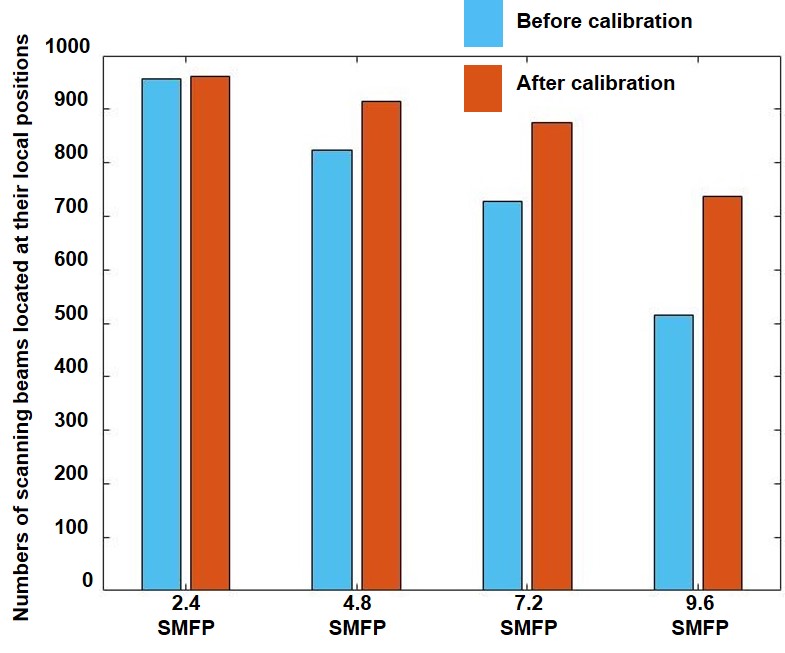


FIG. S3. The effect of wavefront shaping in calibrating the beams.

**2.3 The matched wavefronts and the corresponding simulation beams for focusing point (6,6), (16,16) and (26,26) at 14.4 SMFP**


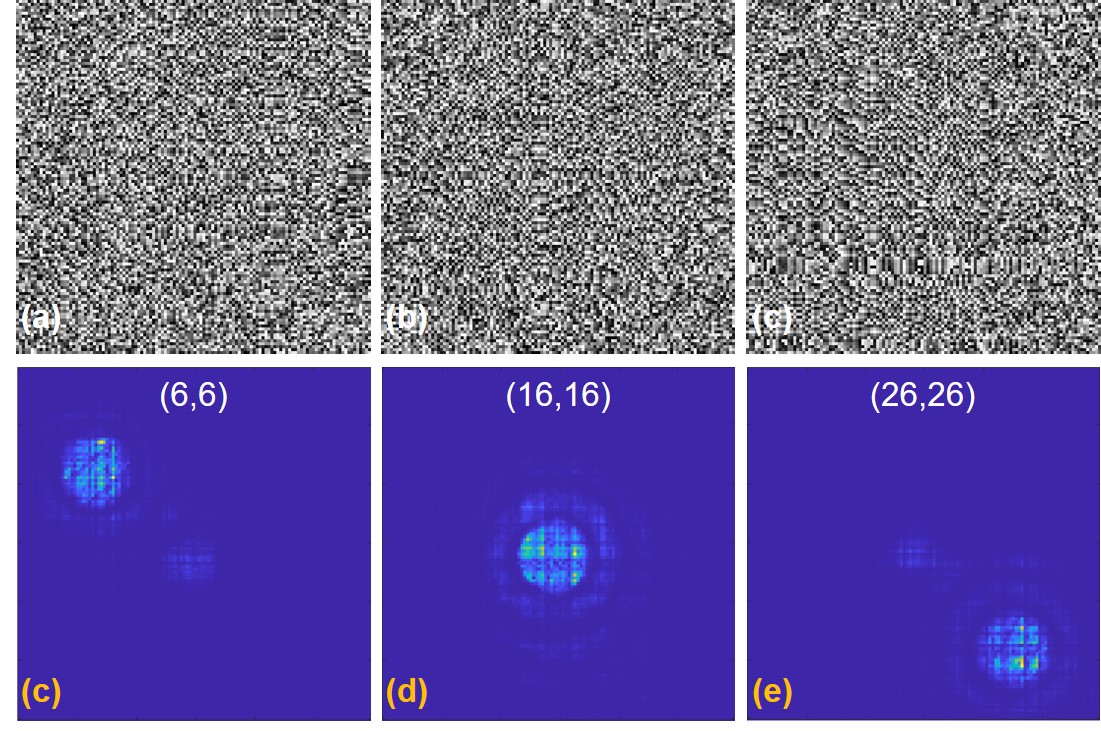


FIG. S4. The simulation beams and their phase patterns.

FIG.S4 (a)-(c) are the calculation wavefronts by our reflection matrix to get the focused beams at (6,6), (16,16) and (26,26), respectively. The values of each pixel ranges from 0 to 2π. At the same time, we also show the simulation results at points at (6,6), (16,16) and (26,26), respectively. Actually, they are similar to the beam intensity at the depth of 14.4 SMFP (as shown in FIG. 3 (h) from the main text).

Reference

1. S. M. Popoff, G. Lerosey, R. Carminati, M. Fink, A. C. Boccara, S. Gigan, "Measuring the transmission matrix in optics: An approach to the study and control of light propagation in disordered media. " Phys. Rev. Lett. 104, 100601 (2010).

2. A. Badon, D. Li, G. Lerosey, A. Boccara, M. Fink, and A. Aubry, “Smart optical coherence tomography for ultra-deep imaging through highly scattering media”, Sci. Adv. 2(11), e1600370 (2016).

3. J. Yang, Y. Miao, T. Huo, Y. Li, E. Heidari, J. Zhu, and Z. Chen, "Deep imaging in highly scattering media by combining reflection matrix measurement with Bessel-like beam based optical coherence tomography", Appl. Phys. Lett. 113, 011106 (2018).

4. Qiang Yang, Jing Cao, Yusi Miao, Jiang Zhu, and Zhongping Chen, "Extended imaging depth of en-face optical coherence tomography based on fast measurement of a reflection matrix by wide-field heterodyne detection," Opt. Lett. 45, 828-831 (2020).
